# Relapsed Type II Lepromatous Reaction With Drug-Induced Hemolysis: A Case Report Highlighting Therapeutic Challenges

**DOI:** 10.7759/cureus.110585

**Published:** 2026-06-10

**Authors:** Mohamed A Baghi, Aiyat Salah Gaffar Mohamed, Maiada Hassan, Suha Babikir, Manoj K Varghese

**Affiliations:** 1 Clinical Department, Weill Cornell Medicine - Qatar (WCM-Q), Doha, QAT; 2 Clinical Department, Qatar University, Doha, QAT; 3 Internal Medicine, Hamad General Hospital, Doha, QAT; 4 Pathology, Hamad Medical Corporation, Doha, QAT; 5 Infectious Diseases, Hamad Medical Corporation, Doha, QAT

**Keywords:** dapson, hansen’s disease, lepromatous leprosy, mycobacterium leprae, type ii lepra reaction

## Abstract

Leprosy is a chronic infectious disease caused by *Mycobacterium leprae* (ML), and it is also known as Hansen’s disease. It is transmitted mainly through prolonged close contact with an untreated infected person. The disease primarily affects the skin and peripheral nerves. Leprosy can, however, present with a broad spectrum of clinical manifestations depending on the host's immune response, ranging from localized tuberculoid disease to disseminated lepromatous disease. Diagnosis of leprosy relies primarily on clinical findings and is supported by histopathological examination of a skin biopsy. Management requires multidrug therapy (MDT), treatment of lepra reactions, and rehabilitation. We report the case of a 27-year-old female with lepromatous leprosy complicated by a severe type two lepra reaction involving multiple organ systems. The treatment regimen was complicated by hemolysis caused by dapsone therapy despite normal glucose-6-phosphate dehydrogenase (G6PD) activity. This report highlights significant therapeutic challenges and provides important insights into the management of leprosy reactional states.

## Introduction

Leprosy is a chronic granulomatous infectious disease caused by the acid-fast bacilli (AFB) *Mycobacterium leprae *(ML) and *Mycobacterium lepromatosis* [1]. The disease primarily affects the skin and peripheral nerves. Leprosy has been classified by Ridley and Jopling into distinct categories based on histopathological and immunological characteristics: tuberculoid (TT), borderline tuberculoid (BT), mid-borderline (BB), borderline lepromatous (BL), and lepromatous leprosy (LL) [1]. Toward the lepromatous end of the spectrum (BL and LL), patients exhibit a high bacillary burden with impaired cell-mediated immunity, leading to disseminated cutaneous and systemic disease and increased susceptibility to type two lepra reactions, particularly erythema nodosum leprosum (ENL) [2]. Prolonged close contact with an untreated person, especially via respiratory droplets, is the predominant mode of transmission [2]. The diagnosis relies primarily on clinical examination and is confirmed through a slit skin smear or biopsy [2,3].

This report presents a case of lepromatous leprosy identified through contact tracing, which was complicated by a severe type two lepra reaction. The reaction was successfully managed with high-dose corticosteroids and thalidomide therapy, resulting in marked clinical improvement. However, the patient developed drug-induced hemolysis due to dapsone therapy despite normal glucose-6-phosphate dehydrogenase (G6PD) activity.

## Case presentation

A 27-year-old female from Sudan presented with a four-month history of progressive skin lesions and sensory changes. She had initially noticed multiple hypopigmented skin patches on her face, upper limbs, and trunk, which were non-painful and non-pruritic, associated with a mild reduction in sensation over the affected areas. There was no history of fever, weight loss, night sweats, cough, joint pain, or trauma. She denied any history of diabetes mellitus, systemic hypertension, autoimmune disease, or immunosuppressive drug use. She reported close household contact with her sister, previously treated for leprosy.

**Figure 1:**
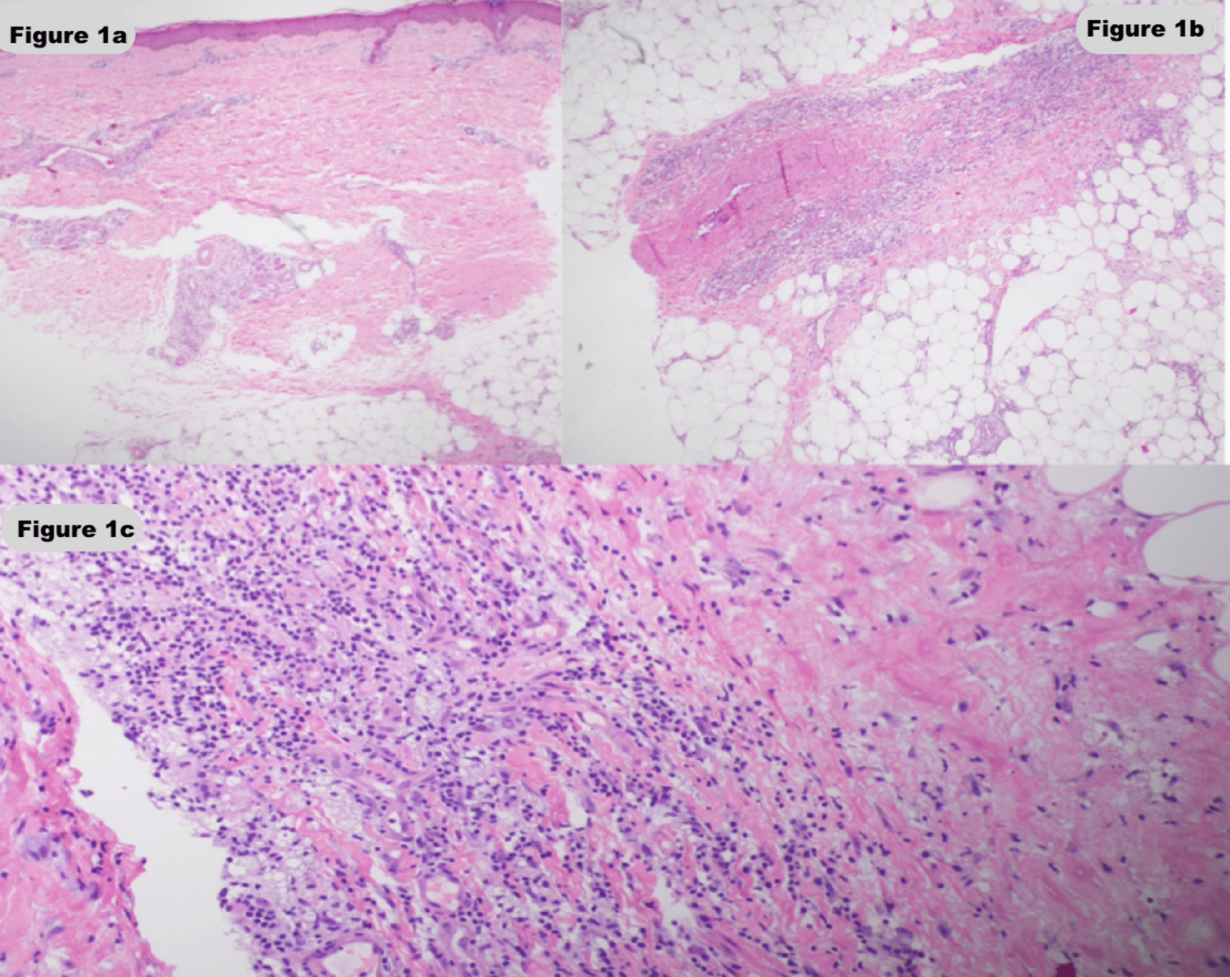
Biopsy findings Figure 1a: Low-power photomicrograph showing epidermal thinning with focal flattening of rete ridges and an underlying relatively unremarkable papillary dermis with mild perivascular inflammation (hematoxylin and eosin stain ×4). Figure 1b: Low-power view of the deep reticular dermis demonstrating ill-defined epithelioid cell granulomas with surrounding lymphocytic infiltrate (hematoxylin and eosin stain ×4). Figure 1c: High-power view highlighting epithelioid histiocytes and lymphocytes forming poorly circumscribed granulomas, without evidence of caseation necrosis (hematoxylin and eosin stain ×60)

On examination, she was hemodynamically stable. Dermatological examination revealed multiple well-defined hypopigmented patches with reduced sensation over the arms and trunk. Peripheral nerve examination showed no significant thickening or tenderness over the ulnar nerves bilaterally. No skin nodules or ulcers were noted. Initial laboratory investigations demonstrated mild microcytic normocytic anemia, with no other biochemical abnormalities (Table 1). The skin biopsy demonstrated focal deep dermal non-caseating granulomas with a periadnexal and perineural distribution (Figure 1), suggestive of leprosy. Based on clinical and histopathological findings, she was diagnosed with BL leprosy. She was referred to an infectious disease specialist who prescribed triple therapy with dapsone, rifampin, and clofazimine.

**Table 1:** Results of laboratory investigations during hospitalization and follow-up MCV: mean corpuscular volume; MCHC: mean corpuscular hemoglobin concentration; LDH: lactate dehydrogenase; ANA CTD: anti-nuclear antibody connective tissue diseases; HIV: human immunodeficiency virus; G6PD: glucose-6-phosphate dehydrogenase; Ab: antibodies; IL: interleukin

Laboratory variables	Day 1 (outpatient)	4th week follow-up (outpatient)	Hospitalization	6-week follow-up	Reference range
White blood cell count (x10³/uL)	10.6	14	5.2	9.5	4 - 10
Hemoglobin (g/dL)	10.9	8.8	11.4	10.6	13.0 - 17.0
MCV (fL)	80.7	84	87.8	86.5	83 - 101
MCHC (gm/dL)	33	32	31	32.3	31.5 - 34.5
Red blood cell count (x10⁶/uL)	4.1	3.3	4.2	3.8	3.8 - 5.5
Hematocrit (%)	33	27	36.8	32.8	40 - 50
Platelet count (x10³/uL)	377	423	392	401	150 - 400
Retic (x10/uL)	-	209	52	90	50 - 100
Peripheral blood smear	-	Schistocytes/anisocytosis	-	-	-
Haptoglobin (mg/dL)	-	173	122	-	30 - 200
Alanine aminotransferase (U/L)	20	10	22	19	0 - 41
Aspartate aminotransferase (U/L)	16	13	15	10	0 - 40
Bilirubin T (umol/L)	4	29	7	11	0 - 21
Bilirubin D (umol/L)	3	10	3	4	0 - 5
Alkaline phosphatase (U/L)	88	101	90	79	40 - 129
Creatinine (mg/dL)	1.44	1.6	1.5	0.9	0.5 - 1.5
Urea (mmol/L)	3.1	5.7	4.6	3.6	2.8 - 8.1
Sodium (mmol/L)	136	135	139	141	135 - 145
Potassium (mmol/L)	4.2	4	3.7	3.6	3.5 - 5.1
Calcium (mmol/L)	2.32	-	-	-	2.12 - 2.60
LDH (U/L)		255	199	-	135 - 214
G6PD screen		Normal	-	-	-
ANA CTD	Negative	-	-	-	-
HIV Ag/Ab	Negative	-	-	-	-
Hepatitis B surface antigen	Negative	-	-	-	-
Hepatitis C antibodies	Negative	-	-	-	-
Toxoplasma Ab IgM	-	-	Negative	-	-
Treponema pallidum Ab	Negative	-	-	-	-
IL6 (pg/mL)	-	-	29	2	< 7

**Figure 2:**
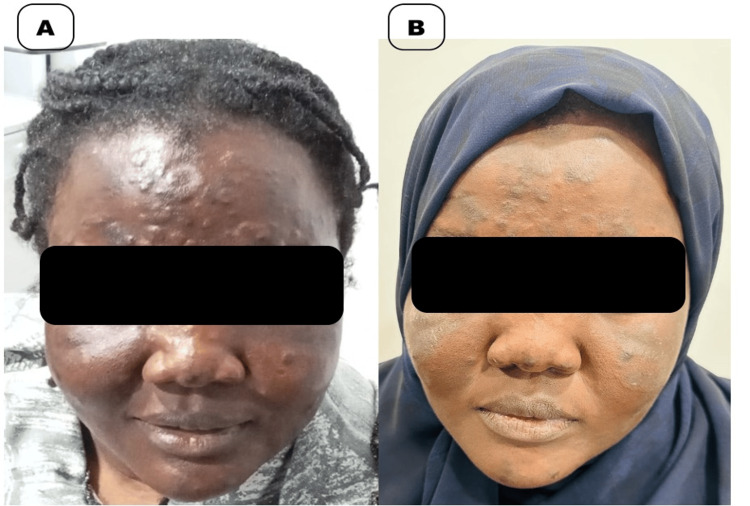
Patient images during the treatment course A: Corresponds to the second severe relapse of type two lepra reaction, showing multiple hyperpigmented to erythematous papules and nodules distributed symmetrically over the forehead, nose, cheeks, and saddle nose, supporting a lepromatous spectrum. B: Following escalation of immunomodulatory therapy, there is partial flattening of the nodular hyperpigmented lesions with reduced erythema and decreased facial infiltration

Four weeks after initiating treatment, follow-up laboratory testing showed evidence of hemolysis despite a normal G6PD test (Table 1). The patient, however, did not report any clinical symptoms of hemolysis. Consequently, dapsone therapy was discontinued, and she was switched to minocycline. Three weeks later, she developed a type two lepra reaction and was started on high-dose prednisolone (60 mg daily) and thalidomide. She gradually improved and achieved remission on low-dose steroids with continued thalidomide maintenance. Over the subsequent months, the lepra reaction resolved completely. Corticosteroid therapy was tapered gradually over six months and then discontinued. Owing to the severity of the initial reaction, maintenance thalidomide at 50 mg daily was continued, with a planned total duration of one year. Treatment interruption due to thalidomide supply depletion, occurring approximately nine months after therapy initiation, was associated with a severe relapse of type two lepra reaction within weeks. The patient developed multiple painful erythematous nodules across the face (Figure 2A) and extremities. Reinitiation of high-dose prednisolone and thalidomide did not halt disease progression. Reassessment at two weeks revealed further deterioration, including high-grade fever, malaise, visual impairment, and severe inflammatory polyarthritis affecting the small joints of the hands and feet, as well as the ankles, wrists, and elbows. Given the progressive systemic involvement, the patient was admitted for inpatient management. Ophthalmologic evaluation revealed no evidence of uveitis, iridocyclitis, or corneal involvement. CT of the head was unremarkable.

During hospitalization, prednisolone was escalated to 100 mg daily and thalidomide to 300 mg daily due to severe recurrent ENL with systemic manifestations and visual symptoms. Laboratory evaluation demonstrated elevated interleukin-6 (IL-6) levels. Following intensification of immunomodulatory therapy, the patient showed marked clinical improvement, with resolution of fever, polyarthritis, and visual symptoms, and was discharged in stable condition. At subsequent outpatient follow-up visits, she demonstrated continued improvement, with marked regression of the facial nodules (Figure 2B). Prednisolone (100 mg daily) and thalidomide (300 mg daily) were maintained for six weeks, after which prednisolone was gradually tapered over the ensuing months, with a total corticosteroid treatment duration of approximately six months. Thalidomide was subsequently reduced to a maintenance dose of 50 mg daily, with plans for prolonged therapy for more than one year. This cautious tapering strategy was adopted because of the severity, recurrence, and corticosteroid dependence that characterized her previous reactional episodes.

## Discussion

Leprosy is a chronic granulomatous infection caused by ML and remains a significant public health concern in endemic regions. It is characterized by a broad spectrum of clinical and immunological manifestations that reflect the host immune response, ranging from tuberculoid to lepromatous disease. Cutaneous lesions are typically the earliest and most common clinical manifestation [2,3]. BL leprosy is part of the Ridley-Jopling spectrum and is characterized by an unstable immunological state, a high bacillary burden, and fluctuating cell-mediated immunity [4].

Type two lepra reaction, also known as ENL, is a systemic immune complex-mediated hypersensitivity reaction that occurs predominantly in patients with BL or LL and does not result directly from bacillary proliferation [4]. ENL is driven by the deposition of antigen-antibody complexes, complement activation, and the upregulation of pro-inflammatory cytokines [4,5]. Clinically, ENL is characterized by painful erythematous nodules, fever, malaise, neuritis, arthritis, and ocular involvement, and is often precipitated by rapid bacillary killing following the initiation of multidrug therapy (MDT) [5].

Our patient presented with hypopigmented anesthetic skin lesions and non-caseating granulomatous inflammation on histopathology. Clinicopathological findings were consistent with BL, and a history of close household contact further increased the diagnostic likelihood. The patient was initially treated with MDT consisting of dapsone, rifampicin, and clofazimine. During treatment, she developed dapsone-induced hemolysis despite normal G6PD activity, necessitating replacement of dapsone with minocycline, which led to the resolution of hemolysis.

Although hemolysis is classically associated with G6PD deficiency, the disorder is uncommon in women because it is inherited in an X-linked manner. Consequently, severe G6PD enzyme deficiency typically requires pathogenic variants affecting both X chromosomes [6]. Nevertheless, heterozygous women may develop clinically significant hemolysis due to skewed X-chromosome inactivation, particularly following exposure to oxidant drugs such as dapsone [6,7]. Importantly, dapsone-induced hemolysis can also occur in individuals with normal G6PD activity owing to oxidative stress mediated by its N-hydroxylated metabolites, particularly dapsone hydroxylamine [7].

These reactive metabolites cause oxidative injury to erythrocyte membranes and hemoglobin, leading to Heinz body formation, reduced erythrocyte deformability, and predominantly extravascular hemolysis. Additional risk factors include high cumulative doses, potentially in patients with renal dysfunction, a slow acetylator phenotype, interactions with concomitant drugs metabolized via the cytochrome P450 system, or inflammatory states that may impair erythrocyte antioxidant capacity [7,8]. This case highlights the importance of close hematological monitoring during multidrug therapy, since normal G6PD activity does not prevent hemolysis.

In our patient, subsequent inflammatory episodes reflected the immunological instability associated with BL and were suggestive of recurrent ENL. Although the initial episodes responded to corticosteroids and thalidomide, relapses that occurred following corticosteroid tapering were accompanied by nodular skin lesions and systemic manifestations, including blurred vision, polyarthralgia, and headache, indicating a severe reactional state. Escalation of therapy to high-dose prednisolone (100 mg/day) in combination with minocycline, thalidomide, clofazimine, and monthly rifampicin resulted in rapid clinical improvement, supporting an immune-mediated mechanism.

IL-6 is an important pro-inflammatory cytokine implicated in the immune response to BL and may serve as a marker of inflammatory activity and treatment response [9]. In our patient, IL-6 levels showed a downward trend following treatment, paralleling clinical improvement and control of systemic inflammation. Thalidomide remains highly effective for severe or recurrent ENL because of its potent inhibitory effects on tumor necrosis factor-alpha (TNF-α) [10]. Nevertheless, its use requires strict precautions owing to its well-established teratogenicity, particularly in women of childbearing potential.

## Conclusions

This report highlights the therapeutic challenges associated with BL complicated by recurrent type two lepra reaction and dapsone‑induced hemolysis. Household exposure remains a critical epidemiological clue. Dapsone-induced hemolysis can occur despite normal G6PD activity, underscoring the need for careful monitoring and follow-up. Furthermore, recurrent reactional episodes illustrate the immunological instability of BL, requiring timely and, at times, intensified immunomodulatory therapy to achieve sustained remission. In addition, strict adherence to prescribed MDT and follow-up is essential to optimize treatment outcomes, prevent relapse, and minimize the risk of recurrent reactional states and associated morbidity.
